# Hydrogel-Based Sensors for Ethanol Detection in Alcoholic Beverages

**DOI:** 10.3390/s19051199

**Published:** 2019-03-08

**Authors:** Jan Erfkamp, Margarita Guenther, Gerald Gerlach

**Affiliations:** Solid-State Electronics Laboratory, Technische Universität Dresden, 01069 Dresden, Germany; mguenthe@mail.zih.tu-dresden.de (M.G.); gerald.gerlach@tu-dresden.de (G.G.)

**Keywords:** stimuli-responsive hydrogel, piezoresistive pressure sensor, ethanol, alcohol, chemical sensor, sensor characterization, ethanol sensitivity, detection limit, cross sensitivity

## Abstract

A fast and reliable determination of the ethanol concentration is essential in the analysis of alcoholic beverages. However, different factors like pH value or salt concentration can influence the ethanol measurement. Furthermore, analytical figures of merit for the alcohol sensor, such as limit of detection, sensitivity and measurement uncertainty, are necessary for the application. In this paper, a detailed sensor characterization of a novel sensor based on ethanol-sensitive poly acrylamide hydrogels will be presented. The resulting swelling pressure of the hydrogel was transformed via a piezoresistive pressure sensor into a measurable output voltage. These kinds of sensors can be used over a large measuring range, up to 50 vol% ethanol and more, with a high sensitivity. In the range from pH 7.4 to 4, the pH value had no influence on the sensor signal. Higher salt concentrations can slightly influence the measurement. The detection limit amounts to 0.06–0.65 vol% ethanol. The concentration of a vodka sample was determined with a sufficient measuring uncertainty.

## 1. Introduction

The determination of the ethanol concentration is important for numerous industrial and biotechnological processes, such as foodstuffs, cosmetics, or pharmaceuticals [1]. Especially for alcoholic beverages, the accurate ethanol measurement is essential due to the strict regulations of the European Union for the labeling of alcoholic drinks [2]. Methods like hydrometers are often used in brewery industry. However, the alcohol measurement accuracy depends strongly on the expertise of the operator and on the temperature of the sample [3]. Other techniques like high-performance liquid chromatography [4] are often too expensive for small companies. This article focuses on a promising novel sensor approach based on stimuli-responsive hydrogels and piezoresistive pressure sensors for the detection of ethanol. Due to low manufacturing costs, a simple sensor set-up and their in-line process capability, these alcohol sensors have a high potential for industrial applications.

Stimuli-responsive hydrogels are hydrophilic three-dimensional polymer networks that can swell and deswell in dependence on a particular stimulus [5]. Used in combination with a piezoresistive pressure sensor, the resulting swelling pressure of the gel deforms the bending plate of the sensor and changes the resistance of piezoresistors integrated therein [6]. A Wheatstone bridge circuit transforms the resistance change into a measurable output voltage. The resulting change in output voltage depends proportionally on the analyte concentration. 

With this concept, different hydrogel-based piezoresistive sensors could be produced by selecting suitable monomers for the preparation of polymerized gels. For example, temperature sensors have already been developed on the basis of the thermosensitive monomer *N*-isopropylacrylamide [7]. Glucose sensors were demonstrated based on 3-acrylamido phenylboronic acid hydrogels [8]. Furthermore, pH-sensitive hydrogel sensors in a physiological range could be realized by using a copolymer based on 2-(dimethylamino)ethyl methacrylate and hydroxypropyl methacrylate [9].

In [10], we demonstrated the feasibility of an ethanol sensor based on polyacrylamide hydrogels and showed the swelling characteristics in different alcohol-water mixtures. In this article, a detailed characterization of such hydrogel-based ethanol sensors will be presented with respect to sensitivity, detection limit, cross-sensitivities, and measurement uncertainty. In particular, factors that have a decisive role for practical application, such as the measuring range, cross sensitivities to salt concentrations, and pH values are investigated. Furthermore, calibration curves were recorded and used to determine the ethanol concentration in vodka samples.

## 2. Materials and Methods

### 2.1. Synthesis of Ethanol-Sensitive Polyacrylamide-Bisacrylamide Hydrogels

**Figure 1:**
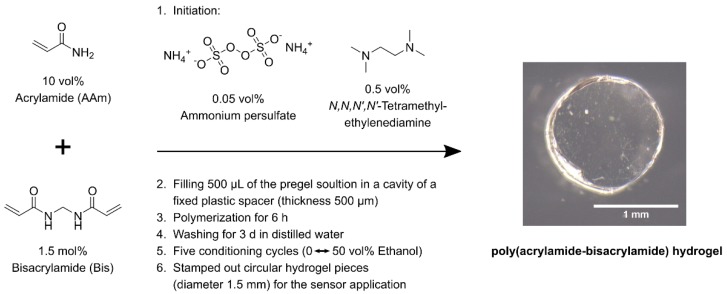
Synthesis of alcohol-sensitive poly(acrylamide-bisacrylamide) hydrogels and their preparation for the application in a piezoresistive pressure sensor.

For the polymerization, 10 vol% (0.95 mmol, 67.83 mg) of the monomer acrylamide (Sigma Aldrich, St. Louis, MO, USA) was combined with 1.5 mol% (14.31 μmol, 2.21 mg) of the crosslinker bisacrylamide (Carl Roth, Karlsruhe, Germany) and were dissolved in distilled water. The total volume of each synthesis was 600 µL. After degassing the pregel solution for 5 min with nitrogen, the hydrogels were polymerized by adding the thermal initiator systems ammonium peroxodisulfate (0.05 vol%, 2.60 μmol, 0.59 mg, Sigma Aldrich, St. Louis, MO, USA) and *N,N,N′,N′*-tetramethylethylenediamine (0.5 vol%, 3.0 µL, Carl Roth, Karlsruhe, Germany). In order to produce sensors with high reproducibility, hydrogels with a defined thickness were synthesized. For this purpose, a plastic spacer with a thickness of 500 µm was fixed between two object slides. The initiated pregel solutions were filled in the cavity of the plastic spacer and were polymerized for 6 h. To remove unreacted monomers, the gels were washed for three days in distilled water. Due to a conditioning process, polymer chains find their optimal arrangement and the measurement accuracy of the sensors increases significantly [11]. For this reason, the gels were conditioned five-fold in an ethanol-water-mixtures (0 vol% ethanol ⇆ 50 vol% ethanol) for a total of five days [10]. The hydrogel synthesis and its preparation for sensor application is shown in Figure 1.

### 2.2. Preparation of Hydrogel-Based Piezoresistive Ethanol Sensors 

**Figure 2:**
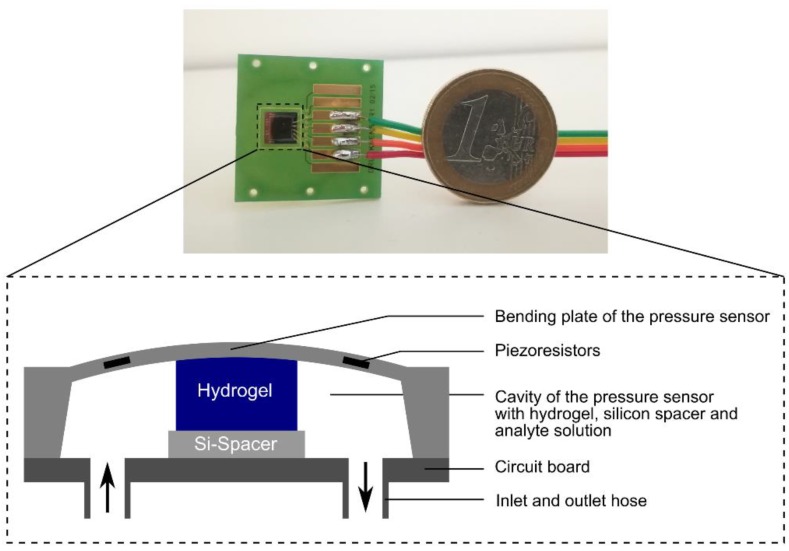
Set-up of a hydrogel-based piezoresistive ethanol sensor: The swelling pressure of the gel leads to a deformation of the bending plate and the piezoresistors. The change in resistance due to the piezoresistive effect will be transformed into an output voltage via a Wheatstone bridge circuit. The figure was modified according to [10].

For the preparation of hydrogel sensors, the thin conditioned gel pieces were used to punch out circular hydrogels with a diameter of 1.5 mm. Meanwhile, a silicon spacer with a thickness of approx. 280 μm was fixed with cyanoacrylate in the middle of a circuit board. A circular hydrogel piece was put on the silicon spacer. Afterwards, the pressure sensor chip (TDK Electronics, previously EPCOS, C41-Series, 5.0 × 5.0 × 0.4 mm^3^, Munich, Germany) was fixed by using cyanoacrylate. This ensured that the hydrogel was located centrally in the cavity of the sensor. After wire bonding providing a signal transmission between sensor and circuit board, the connection cables to the read-out unit (Fluke 45 Multimeter, Glottertal, Germany) were soldered. The inlet and outlet hoses were fixed with cyanoacrylate and two-component epoxy resin on the circuit board. The tubes and a peristaltic pump (Reglo Digital MC-2/6, Ismatec as part of Cole-Parmer, Wertheim, Germany) were used to pump ethanol–water mixtures to the sensor at a constant flow rate of 0.2 mL/min. The supplied voltage of the ethanol sensor was 5 V (DIGI 35, Voltcraft as part of Conrad Electronic, Hirschau, Germany). All sensor measurements were done at room temperature [10]. The resulting hydrogel-based ethanol sensor is shown in Figure 2. For the different measurement tasks, new sensors were always made. The nomenclature of the used hydrogel-based ethanol sensors was summarized in Appendix A. 

### 2.3. Determination of the Measuring Range

After the sensor preparation, the hydrogel sensor was conditioned overnight in distilled water. Ethanol–water mixtures between 10 and 50 vol% ethanol with 10 vol%-steps and distilled water were prepared. During the measurements, mixtures were changed to the next higher ethanol concentration every 90 min.

### 2.4. Cross-Sensitivity to Salt Concentrations

The influence of the salt concentration on the sensitivity of the sensors was exemplarily demonstrated using sodium chloride solutions. Sodium chloride solutions with a concentration of 0.01, 0.05, 0.1, and 0.5 mol/L were prepared using distilled water. First, all sensors were conditioned overnight in distilled water. During the measurement, the solutions were changed every 90 min to the next higher sodium chloride concentration. Finally, a solution with 20 vol% ethanol and 0.5 mol/L sodium chloride was made and measured with the sensors.

### 2.5. Cross-Sensitivity to pH Value

The cross-sensitivity to the pH value was tested with phosphate-buffered saline (PBS). One PBS tablet (pH 7.4, I = 0.15 mol/L in 200 mL water, Sigma Aldrich, St. Louis, MO, USA) was dissolved in 200 mL distilled water. The pH value of the solutions was adjusted to pH 6.02, 4.97, and 4.01 using a pH meter and 0.1 mol/L hydrochloric acid. After conditioning the sensors overnight in PBS buffer, pH 7.4, the buffer solutions were measured and changed to the next lower pH value every 90 min. At the end, a PBS-buffer solution, pH 4.01, with 20 vol% ethanol was produced and also measured with the sensors. 

### 2.6. Sensor Calibration and Measurements of a Vodka Sample

To determine the alcohol content of vodka (brand: “Wodka Gorbatschow” with 37.5 vol% ethanol, Henkell & Co., Sektkellerei KG, Wiesbaden, Germany), a calibration curve was prepared first. For this purpose, ethanol–water mixtures with 34, 36, 38, and 40 vol% ethanol were made and each sample was measured for 90 min. Subsequently, the vodka sample was analyzed with the help of the derived calibration curves and the alcohol concentration of the vodka could now be determined.

## 3. Results and Discussion

### 3.1. Determination of the Measuring Range

**Figure 3:**
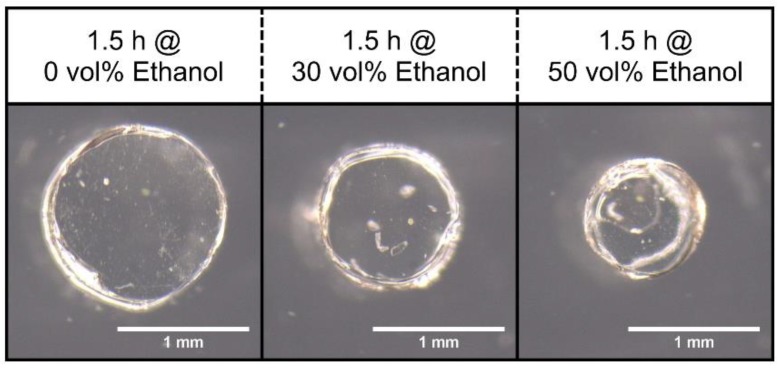
Swelling of a poly(acrylamide-bisacrylamide) hydrogel dot (diameter ca. 1.5 mm) in different ethanol–water mixtures.

Most alcoholic beverages have an alcohol content of up to 50 vol% ethanol. For this reason, the sensitivity was studied in a range from 0 to 50 vol% ethanol. At first, the hydrogel swelling of a hydrogel dot was tested in different ethanol–water mixtures via microscopy (Figure 3).

**Figure 4:**
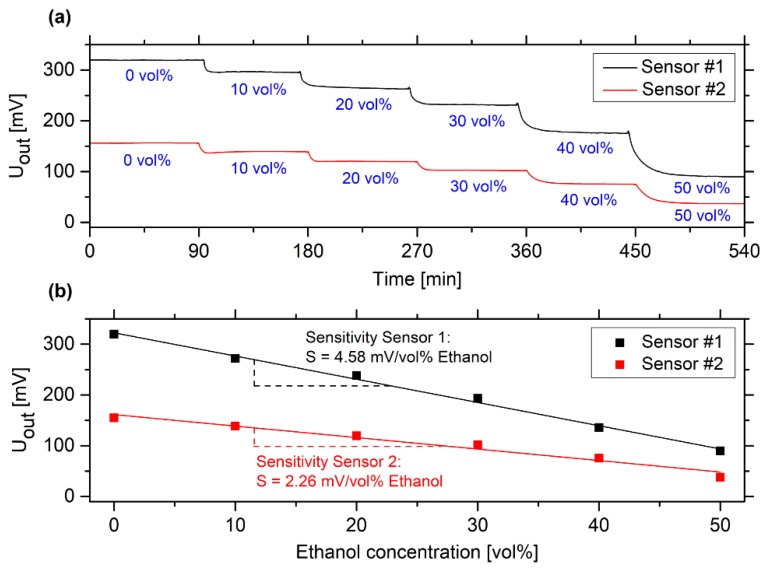
(**a**) Time-dependent change of the output voltage for two hydrogel-based ethanol sensors at different ethanol concentrations in a range from 0 to 50 vol% ethanol; and (**b**) the corresponding calibration curves of these sensors.

Polyacrylamide hydrogels deswell with increasing ethanol concentrations in a wide measuring range. In this context, the solubility of the polymer system was of decisive importance. Polyacrylamide is soluble in water; however, the polymer is insoluble in ethanol. In water, the solvent interacts with the polymer chains and the hydrogel is swollen. In ethanol, the hydrogel chains do not interact with the solvent and, hence, the hydrogel is deswollen [10]. Since the swelling depends on the ethanol concentration, these hydrogel systems are excellently suited to be used in hydrogel-based piezoresistive ethanol sensors (Figure 4). 

In comparison to [10], hydrogels with a higher linker content (1.5 mol% instead of 0.44 mol% bisacrylamide) were used in this application. The composition of the hydrogel influences significantly the sensitivity of the sensor. The higher the concentration of the linker, the more crosslinking points are in the gel. As a result, the hydrogels have a lower swelling degree [10,12,13], which should indicate a lower sensitivity of the gel sensors. However, the swelling pressure of the gel is decisive for the sensitivity of such hydrogel-based pressure sensors. Here, the mechanical stability, in particular the Young’s modulus, is particularly relevant. Due to the higher linker concentration and the resulting enhanced number of crosslinking points, the Young’s modulus will be increased [14]. This leads to a better mechanical stability in the sensor application [15]. 

All alcohol sensors were highly sensitive to the ethanol concentration over a wide measuring range, from 0 up to 50 vol% ethanol. The sensor characteristic in the investigated measuring range was almost linear. However, both sensors showed different sensitivity values (4.58 and 2.26 mV/vol% ethanol). We assume that this was caused by inhomogeneities during the polymerization of the gel, that lead to areas with higher or lower monomer concentration in the solution and, hence, to a locally varying number of crosslinking points, whereby the elastic properties can vary [14]. The manual assembly of the sensor also influences the sensitivity. If the hydrogel is not placed centrally in the cavity of the pressure sensor or if the gel moves during swelling, force transmission to the bending plate of the pressure sensor can be changed and, thus, influence the sensor signal. In order to minimize this effect, more precise assembly methods should be used for industrial applications. At the moment, each hydrogel-based sensor has to be calibrated individually due to these large deviations in sensitivity. 

### 3.2. Cross-Sensitivity to Salt Concentrations

**Figure 5:**
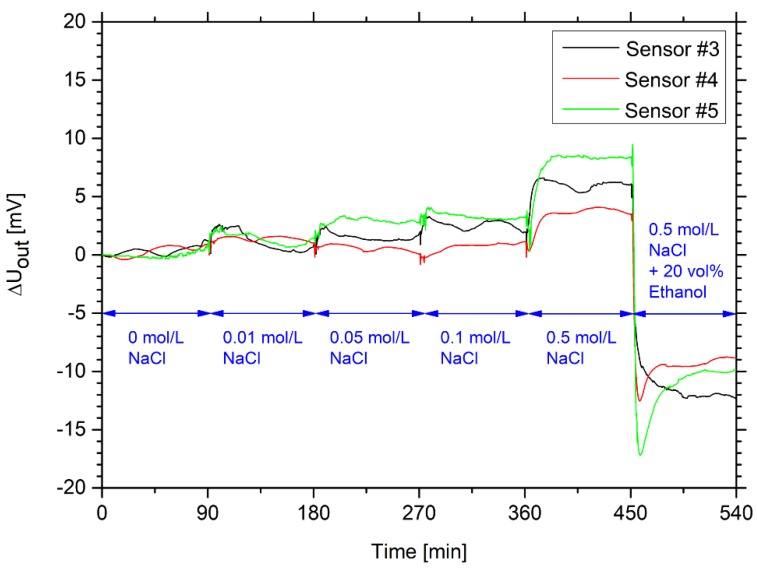
Time-dependent change of the output voltage for three hydrogel-based sensors in ethanol-free solutions with different values of the ionic strength, in a range from 0.01 to 0.5 mol/L. To underline the ethanol sensitivity of the sensors, a solution with 20 vol% ethanol and 0.5 mol/L sodium chloride was measured at the end of the experiment.

In alcoholic beverages, there are many different ions due to the used drinking water. In the brewing industry, the addition of carbon dioxide has also an influence on the ionic strength of the sample. However, the ionic concentration in the sensor application could also have a significant influence on the swelling properties depending on the chemical composition of the hydrogels [16,17,18]. This could influence, negatively, the ethanol measurement. For this reason, the influence of the ionic strength was tested with different sodium chloride solutions (Figure 5). With increasing salt concentration, the hydrogel swelled significantly. Sivanantham and Tata investigated the swelling properties and the polymer–solvent interaction parameter of polyacrylamide hydrogels in sodium chloride solutions [19]. The polymer–solvent interaction parameter decreased with increasing the salt concentration. This led to a decrease in polymer–polymer affinity due to electrostatic double-layer formation of the sodium and chloride ions with the polar groups of poly acrylamide (C=O and C–N). Due to the increasing salt concentration and the resulting decrease of polymer–polymer interactions, poly acrylamide swells in sodium chloride solutions [19]. Similar effects can also be expected with other ionic species. 

In the sensor measurements, a significant swelling of the hydrogels was detected for salt concentration with equal or more than 50 mmol/L sodium chloride. For these sensors, baseline noise was determined of maximum 1 mV. This uncertainty of measurement is significantly influenced due to the pulsation of the used peristaltic pump to generate a constant flow rate. This uncertainty could be minimized by using a low-pulsation gear pump [20]. Other noise sources, like air bubbles in the sensor system due to dissolved gases in the liquid, can also influence the measuring uncertainty. Other uncertainty factors, such as the background noise of the pressure sensor itself, can be neglected.

In general, drinking water contains many different cations and anions. In practice, for comparing the results of samples with different ion species, the total equivalent concentration is usually used in drinking water analytics. Here, a differentiation was made between the cation- and anion-equivalent concentration, respectively. For example, the cation-equivalent concentration could be calculated as the sum of the product of the absolute value of the charge of a cation species *z_i,cat_* and its concentration *c_i,cat_* over all cation species *N_cat_* in the sample (Equation (1)). The total anion-equivalent concentration was determined similarly [21,22]. The total anion-equivalent concentration must be equal to the total cation-equivalent concentration due to the electron neutrality in the solution [22,23].

(1) $$c_{eq}=\overset{N_{cat}}{\underset{i}{\sum }}c_{i,cat}\left|z_{i,cat}\right|=\overset{N_{an}}{\underset{j}{\sum }}c_{j,an}\left|z_{j,an}\right|$$

In 2018, drinking water in Dresden (Germany) had total cation- and anion-equivalent concentrations between 2.4 and 6.8 mmol/L [24]. In case of the tested sodium chloride solutions, these values corresponded directly to the concentration of the salt solution. The lowest tested salt concentration (10 mmol/L) contained up to four times higher total ion-equivalent concentration in comparison to drinking water. At this concentration; however, no significant change in output voltage could be observed. Therefore, the cross-sensitivity of the ions in drinking water could be neglected. 

### 3.3. Cross-Sensitivity to pH Value

**Figure 6:**
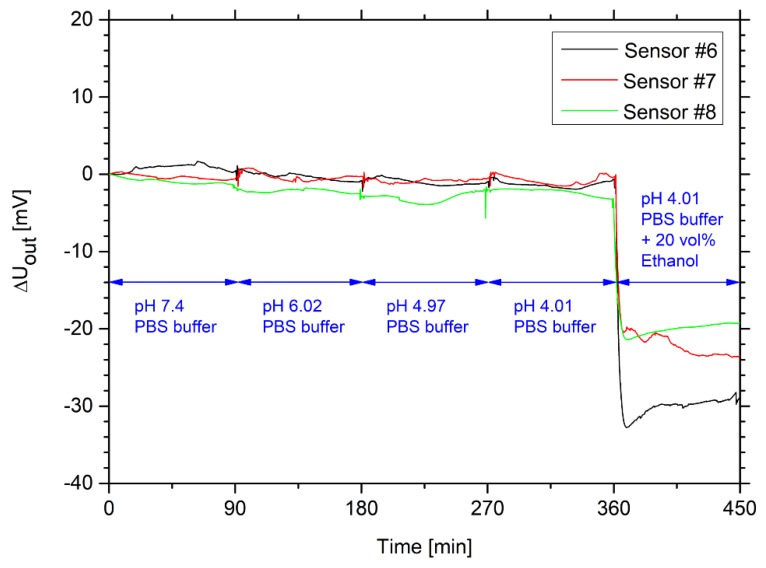
Time-dependent change of the output voltage for three hydrogel-based sensors in ethanol-free PBS solution with different pH values in a range from pH 7.4 to 4.01. To underline the ethanol sensitivity of the sensors, a 20 vol% ethanol solution with PBS buffer, pH 4.01, was measured at the end of the experiment.

Many different alcoholic beverages have often an acidic pH value. For example, due to the addition of carbonic acid in beer, the pH value of beer lies usually in a range between pH 4 and 4.5 [25,26]. Sweet liquors have a pH value in a range from pH 3.3 to 3.9, and strong alcoholic drinks in a range from pH 6.5 to 6.9 [27]. However, most hydrogel systems show an influence of the pH value on the swelling behavior [28,29,30]. Therefore, the pH sensitivity of hydrogel-based ethanol sensors was tested in an acidic pH range from pH 7.4 to 4.01 (Figure 6). 

All sensors showed almost no cross-sensitivity to pH in this range. The baseline noise of less than 1 mV was caused by the same reason as described in Section 3.1. Polyacrylamide is a neutral polymer [31]. In comparison to other pH-sensitive hydrogels, this polymer system has no charged functional groups. This eliminates the repulsive interactions of charged side groups that often explain the pH-dependent swelling of hydrogels [32,33,34]. As a result, neutral polyacrylamide hydrogels do not swell as a function of the pH value. Nesrinne and Djamel have studied the pH-dependent swelling properties of polyacrylamide hydrogels and found no significant influence in the range from pH 2 to 10 [35]. Therefore, the influence of the pH value for the ethanol sensor measurement could also be neglected.

### 3.4. Calibration Curves and Measurement of a Vodka Sample

**Figure 7:**
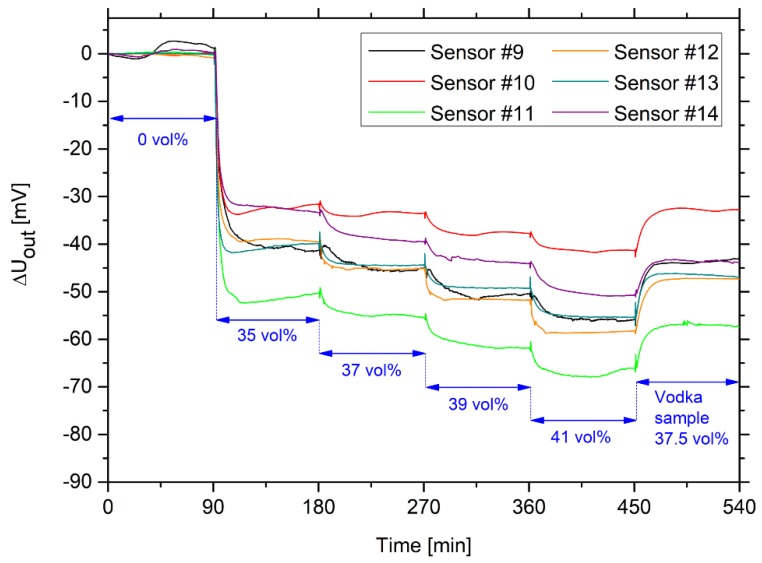
Time-dependent change in output voltage for six hydrogel-based sensors in ethanol–water mixtures with defined ethanol concentrations (between 35 and 41 vol% ethanol) for the preparation of calibration curves. At the end of the experiment, a vodka sample (“Wodka Gorbatschow” with 37.5 vol% ethanol) was measured to calculate the ethanol concentration of the vodka using the calibration curves.

The potential of hydrogel-based ethanol sensors for industrial applications was demonstrated exemplarily by determining the alcohol concentration in a vodka sample (“Wodka Gorbatschow”, Henkell & Co., Sektkellerei KG, Wiesbaden, Germany) with a given value of 37.5 vol% ethanol. For the preparation of calibration curves, ethanol–water mixtures with a known ethanol concentration and, furthermore, the vodka sample were prepared and measured with the sensors (Figure 7).

**Figure 8:**
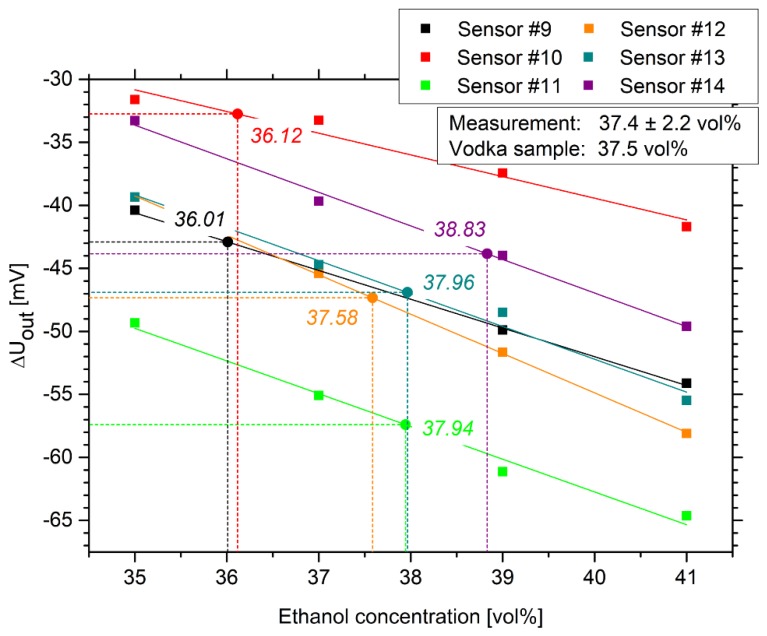
Calibration curves for the hydrogel-based ethanol sensors used for the determination of the ethanol concentration of a vodka sample (“Wodka Gorbatschow” with 37.5 vol% ethanol).

Based on these measurements, an individual calibration curve was prepared for each sensor. Each sample was measured for 90 min until the output voltage was nearly constant. The change in output voltage was plotted as a function of the ethanol concentration. Based on the fitted linear regression curves, the corresponding ethanol concentrations were determined for the measured vodka sample. The measured ethanol concentration is given for each sensor in Figure 8. Furthermore, the uncertainty in measurements was calculated according to the ISO/IEC Guide 98-3: “Guide to the expression of uncertainty in measurement” (GUM) [36]. After the calculation of the mean value and the standard deviation of the determined ethanol concentrations, the standard deviation was then multiplied by the coverage factor *k* = 2 to ensure a confidence probability of 95%. The results were given as the mean value plus-minus the doubled standard deviation in Figure 8. 

All tested sensors also had a linear calibration curve in the used measuring range, as shown in Figure 4. The sensitivity (see Appendix B) of the ethanol sensors was between 1.72 and 3.13 mV/vol% ethanol. The ethanol concentration of the vodka sample was determined as 37.4 vol% within a standard deviation of 2.2 vol%. According to the manufacturer, the investigated vodka sample has an ethanol concentration of 37.5 vol% ethanol, which is almost identical to the sensor measurements. As shown in Section 3.2 and Section 3.3, the pH value and the salt concentration of the sample did not influence the sensor measurement significantly. 

As the measurement results show, hydrogel-based ethanol sensors could be interesting for the usage of ethanol detection in alcoholic beverages. This is particularly due to their advantages, namely the fast sensor response, the low-cost of the sensors, their inline capability, their low cross sensitivities to the pH value or salt concentrations, their low detection limits, and their reliable detection of the ethanol concentration in a vodka sample. Only the measurement uncertainty of the sensors needs to be improved for reliable detection in industrial application. Here, an automated sensor fabrication instead of a manual assembly should lead to a significant improvement of the measurement uncertainties. 

### 3.5. Limit of Detection (LoD) and Limit of Quantification (LoQ)

During the measurements, it was possible to detect concentration changes of down to 2 vol% ethanol for all sensors. This means that the limit of detection (*LoD*) and the limit of quantification (*LoQ*) must be below this value. For the determination of the detection and quantification limit in detail, the German standard specification DIN 32654: “Chemical analysis—Decision limit, detection limit and determination limit under repeatability conditions—Terms, methods, evaluation” [37] was used. 

The *LoD* is defined as the lowest analyte concentration that can be detected in the sample. Based on the *LoD*; however, only a qualitative statement can be made as to whether the solution contains the analyte or not. For a quantitative analysis, the *LoQ* can be used which is the lowest analyte concentration at which a certain measuring value can be specified. According to DIN 32654, the *LoD* and the *LoQ* can be calculated as:

(2) $$x_{LoD/LoQ}=f_{LoD/LoQ}\cdot \frac{s_{L}}{b}$$

where *s_L_* is the standard deviation of the sample without the analyte and *b* the slope of the calibration curve (sensitivity). The factor *f* was calculated individually for the *LoD* (*f_LoD_* = 3.30) and *LoQ* (*f_LoQ_* = 5.90), respectively. The detailed calculations for these factors according to DIN 32654 are shown in Appendix B.

The *LoD* lay, for all sensors, in a range from 0.06 to 0.65 vol% ethanol, whereas the *LoQ* amounted to 0.10 to 1.17 vol% ethanol. An exception was Sensor #1, which had a significantly higher *LoD* (2.11 vol% ethanol) and *LoQ* (3.78 vol% ethanol), respectively. However, as shown in Figure 7, changes in the ethanol concentration of 2 vol% could already be resolved. This means that the *LoQ* and also the *LoD* must be lower than 2 vol%. For the determination of the *LoD* and *LoQ*, respectively, the signal noise of the sample without ethanol plays a decisive role. Different factors, like air bubbles due to dissolved gases in the sample, can be responsible for this larger deviation of this measurement, which increases the *LoD* and *LoQ* according to Equation (2). Like the measurement uncertainty, the *LoD* and the *LoQ* could also be decreased by automated sensor production.

## 4. Summary

In this work, we presented hydrogel-based piezoresistive ethanol sensors and tested their properties for a possible application for the detection of ethanol in alcoholic beverages. The sensor based on the ethanol-sensitive poly acrylamide is highly sensitive to typical ethanol concentrations in alcoholic drinks in the range of up to 50 vol% ethanol. Even small changes in the alcohol concentration can be detected due to the low *LoD* and *LoQ*, respectively. Factors like salt concentration or pH value of the sample have no significant influence for the sensor application. Furthermore, the ethanol concentration of a vodka sample (“Wodka Gorbatschow”) with a given value of 37.5 vol% ethanol was determined as 37.4 ± 2.2 vol% ethanol. In comparison to commercially available sensor systems and further sensor concepts in research and development (Table 1), hydrogel-based piezoresistive ethanol sensors could be an alternative for the determination of the ethanol concentration in alcoholic beverages.

## Figures and Tables

**Table 1:** Ethanol sensor concepts and their advantages and disadvantages—a comparison.

Method	Advantages (+) and Disadvantages (−)	
Chromatographic methods [38,39]	(+)	Most sensitive and accurate method [40,41]
	(**−**)	Very high acquisition and operating costs [40,41], especially for smaller companies
	(**−**)	Well-trained operator necessary due to difficult handling of the method [40]
Optical sensors [42,43]	(+)	Wide fields of application due to large measuring ranges (2–50 vol% [42], 5–50 vol% [43])
	(**−**)	High *LoD* (1.5 vol% [42], 2 vol% [43])
	(**−**)	Significant cross-sensitivity to pH [42]
	(**−**)	Dye leaching over time possible [41]
Microbial [44] and enzymatic [45] biosensor	(+)	Measuring range: 0.05–5 mmol/L [44], 0.1–5 mmol/L [45], after dilution also usable for alcoholic beverages [44,45]
	(**−**)	Microbial and enzymatic activity depends on different factors (e.g., temperature [44,45], pH [45])
	(**−**)	Poor long-term stability due to loss of microbial and enzymatic activity over time [44,45]
Hydrogel-based sensor (presented in this work)	(+)	Wide measuring range (up to 50 vol%)
	(+)	Low *LoD* (0.060–0.56 vol%)
	(+)	No relevant salt or pH cross-sensitivity
	(+)	Low-cost sensor (~10€/Sensor)
	(+)	Small size, even more miniaturizable
	(+)	In-line process capability
	(**−**)	Measurement uncertainty must be improved

## Appendix A. Nomenclature of the Used Hydrogel-Based Ethanol Sensors

Various hydrogel-based ethanol sensors were used for different research topics. Therefore, all used sensor and their measuring applications are summarized in Table A1.

**Table A1:** Overview of all hydrogel-based ethanol sensors including their measurement tasks.

Sensors	Sensors Were Used for
Sensor #1–#2	Determination of the measuring range (Section 3.1)
Sensor #3–#5	Cross-sensitivity to different salt concentrations (Section 3.2)
Sensor #6–#8	Cross-sensitivity to different pH values (Section 3.3)
Sensor #9–#14	Preparation of calibration curves, measurement of vodka samples (Section 3.4), and calculation of the *LoD* and *LoQ* (Section 3.5)

## Appendix B. Determination of the Limit of Detection (*LoD*) and Limit of Quantification (*LoQ*) according to DIN 32654

### Appendix B.1. Limit of Detection (LoD)

The detection limit is the lowest analyte concentration at which the analyte can be detected in the sample with a certain error probability for the Type I (*α*) and Type II (*β*) error. The *LoD* for a single measurement of the analytical sample can be estimated according to the following equation:

(A1) $$x_{LoD}=2\cdot t_{f;\alpha }\cdot \sqrt{1+\frac{1}{n}}\cdot \frac{s_{L}}{b}.$$

Here *t_f;α_* is the quantile of the one-tailed Student’s *t*-test, *f* the degree of freedom (*f* = *n* − 1), *α* the probability for the Type I error, *n* the number of measurements of the sample without the analyte, *s_L_* the standard deviation of the sample without the analyte, and *b* the slope of the calibration curve (sensitivity).

In all cases, the error probability amounts to 5% (*α* = *β* = 0.05). Each analyte solution, including the solutions without ethanol, was measured for 90 min where the measurement signal was recorded every 10 s. However, the last measuring points must be neglected for the determination of the standard deviation *s_L_* due to sensor signal fluctuations during solvent changes. For this reason, only the first 500 measuring points (*n* = 500) were considered for the calculation. Due to the large number of measuring points, the degree of freedom can be simply equated with the number of measuring points (*n* ≈ *f* = 500). The quantile of the one-tailed Student’s *t*-test (*α* = 0.05, *f* = 500) is 1.648 [46]. Inserting the known values in Equation (A1) yields:

(A2) $$x_{LoD}=\;3.30\cdot \frac{s_{L}}{b}$$

The standard deviation *s_L_* and the slope of the calibration curve *b* for each sensor were determined by using the program Excel. Afterwards, the *LoD* was calculated for each sensor by using Equation (A2). The calculated results for the *LoD* as well as for the *LoQ* are presented in Table A2.

### Appendix B.2. Limit of Quantification (LoQ)

The limit of quantification is the lowest analyte concentration at which a specific certain value with a certain error probability can be specified. Above the *LoD* but below the *LoQ*, the analyte was identified in the sample; however no analyte content may be specified. An indication of the content is only possible above the *LoQ* value. For a single measurement of the analytical solution, the *LoQ* can be estimated as:

(A3) $$x_{LoQ}=\;k\cdot t_{f;\frac{\alpha }{2}}\cdot \sqrt{1+\frac{1}{n}}\cdot \frac{s_{L}}{b}$$

where *k* is the relative result uncertainty for characterization of the determination limit (e.g., 33,3% for *k* = 3), *t_f;_*_α*/2*_ the quantile of the two-tailed Student’s *t*-test, *f* the degree of freedom (*f* = *n* − 1), α the probability for the Type I error, *n* the number of measurements of the sample without the analyte, *s_L_* the standard deviation of the sample without the analyte and *b* the slope of the calibration curve (sensitivity).

The relative result uncertainty is freely selectable according to DIN 32654, but a value of 33.3% (*k* = 3) is frequently assumed. The quantile of the two-tailed Student’s *t*-test (*α/2* = 0.05, *f* = 500) amounts to 1.965 [46]. The other values (*α* = *β* = 0.05, *n* = 500) remain the same compared to the calculation of the detection limit. After insertion in Equation (A3), it yields:

(A4) $$x_{LoQ}=\;5.90\cdot \frac{s_{L}}{b}$$

Knowing the standard deviation *s_L_* and the slope of the calibration curve *b*, which have already been calculated to determine the *LoD* value, the *LoQ* can also be calculated by using Equation (A4). The resulting *LoD* and *LoQ* for all tested sensors are shown in Table A2.

**Table A2:** Determination of the Limit of Detection (*LoD*) and Limit of Quantification (*LoQ*) values for all hydrogel-based ethanol sensors in Section 3.4.

Sensor	*s_L_* [mV]	*b* [mV/vol%]	*LoD* [vol%]	*LoQ* [vol%]
Sensor #9	1.46	2.28	2.11	3.78
Sensor #10	0.16	1.72	0.31	0.55
Sensor #11	0.13	2.60	0.17	0.31
Sensor #12	0.17	3.13	0.18	0.32
Sensor #13	0.04	2.61	0.06	0.10
Sensor #14	0.53	2.66	0.65	1.17

*s_L_*: standard deviation of the sample without the analyte, *b*: slope of the calibration curve (sensitivity).
